# Multicenter, prospective cohort study: immediate postoperative gains in active range of motion following robotic-assisted total knee replacement compared to a propensity-matched control using manual instrumentation

**DOI:** 10.1186/s42836-023-00216-0

**Published:** 2023-12-04

**Authors:** Camdon Fary, Jason Cholewa, Anna N. Ren, Scott Abshagen, Mike B. Anderson, Krishna Tripuraneni

**Affiliations:** 1 Epworth Foundation, Richmond, VIC 3121 Australia; 2 Department of Orthopaedics, Western Hospital, Melbourne, 3011 Australia; 3 Zimmer Biomet, Warsaw, IN 46850 USA; 4 New Mexico Orthopaedic Associates, Albuquerque, NM 87110 USA

**Keywords:** Total knee arthroplasty, Patient-reported outcome measures, Knee osteoarthritis, Robotic-assisted surgery

## Abstract

**Background:**

Range of motion (ROM) following total knee replacement (TKR) has been associated with patient satisfaction and knee function, and is also an early indicator of a successful procedure. Robotic-assisted TKR (raTKR) is considered to reproduce more precise resections, and, as a result, may be associated with improved early patient satisfaction compared to manual TKR (mTKR). The purpose of this study was to evaluate the early postoperative active ROM (aROM) between raTKR and mTKR.

**Methods:**

A total of 216 mTKR patients were propensity-matched, in terms of age, gender, comorbidities, and BMI, to 216 raTKR cases. Intraoperative and immediate postoperative adverse events were collected. Knee flexion and extension aROM were measured preoperatively and at one- and three months after operation.

**Results:**

Changes in flexion aROM were significantly greater in raTKR vs. mTKR at one- (6.9°, 95% CI: 3.5, 10.4°) and three months (4.9°, 95% CI: 2.1, 7.7°). Flexion aROM was greater at three postoperative months compared to preoperative aROM only in the raTKR group, and raTKR patients had higher odds of achieving ≥ 90° of flexion at one month after operation (OR: 2.15, 95% CI: 1.16, 3.99). There were no significant differences between groups in intraoperative (*P* > 0.999) or postoperative adverse events.

**Conclusions:**

Compared with mTKR, raTKR resulted  in less loss of aROM immediately after operation and a faster recovery of aROM within three months after operation.

**Trial registration:**

Clinicaltrials.gov (NCT# 03737149).

## Background

For advanced knee osteoarthritis (OA), total knee replacement (TKR) is recognized as a safe and effective treatment to alleviate pain and restore function [1]. Despite progressive advancements over the last thirty years in component technology and surgical techniques, 15%–20% of patients remain dissatisfied with the procedure [2, 3]. Poor alignment and inaccurate prosthesis positioning are thought to contribute to the pain, instability, and range of motion limitations reported during activities of daily living by dissatisfied TKR patients [4, 5].

Robotic-assisted technology has been adopted by surgeons over the last two decades to improve the accuracy of bone resections and the postoperative efficacy of TKR. Numerous studies provided evidence that robotic-assisted TKR (raTKR) can improve implant positioning, limb alignment, and gap balance, [6–9] and lead to better short-term outcomes compared to manual TKR (mTKR) [10–13]. However, not all studies reported superior patient-reported outcome measures (PROMs) of function following raTKR [14–17]. These discrepancies raised questions regarding the utility of subjective PROMs in the full evaluation of the potential functional benefits associated with increased raTKR accuracy [18]. For example, Williams et al. demonstrated a lack of correlation between PROMs and physical function [19], and Nilsdotter et al. reported a significant proportion of patients’ physical activity expectations (i.e., ability to dance or golf) were not met, yet patients reported high satisfaction with regard to both pain relief and physical function on PROMs [20]. These reports suggest that objective assessments should be included when evaluating TKA outcomes.

Knee range of motion (ROM) is a commonly employed objective measure following TKR. Approximately 67° of knee flexion is required during the swing phase of the gait cycle, 90° is needed to ascend/descend stairs, and ROMs well exceeding 90° are necessary to perform recreational activities that involve squatting and kneeling [21, 22]. Passive ROM at 12 to 24 postoperative months has been found to be positively associated with Western Ontario and McMaster University Arthritis Index (WOMAC) [23], Oxford Knee Score (OKS) [24], and the 12-item Short Form (SF12) health and satisfaction scores [19]. Although postoperative ROM (i.e., 5 days after operation) is not associated with mid- to long-term ROM outcomes due to individual differences in pain level, tolerance, and medications [25], several studies suggested that measuring ROM between 1 to 3 months postoperatively is predictive of satisfaction, quality of life and the 12-month ROM [19, 25, 26]. Furthermore, for patients with higher preoperative ROMs (i.e., > 120°), reaching 105°of ROM within one month, appears to be the minimum benchmark for achieving 120° ROM at 12 months [25], suggesting that early restoration of ROM is essential to the achievement of a critical ROM at 12 months whereby activities of daily living may be accomplished.

Differences in ROM outcomes between raTKR and mTKR are ambiguous. Some studies reported greater ROM with raTKR [11, 27, 28], but several others found no difference [14–17, 29]. Two studies reported greater early postoperative (one day to one month) ROMs with raTKR that were not sustained through 6 months to one year [7, 30]. All except one of these studies [28] measured passive ROM; however, active ROM (aROM) may be more indicative of the available ROM to perform the recreational activity and accomplish activities of daily living [22]. The purpose of this study was to evaluate differences in early postoperative active ROM between raTKR and manual TKR (mTKR). Additionally, we reviewed PROMs through one-year postoperative and intra- and postoperative medical events and surgical data.

## Methods

We performed a secondary analysis on data collected from an ethically-approved (WCG IRB # 20182013) global, multicenter prospective cohort study. The clinical study, A Prospective Multicenter Longitudinal Cohort Study of the mymobility Platform [31, 32], was initiated in 2018 and listed on clinicaltrials.gov (NCT# 03737149). Given the purpose of this analysis, we limited our review of the study data to patient demographics, comorbidities, and objective clinical evaluations (ROM). We also reviewed one-, three- and twelve-month Knee Injury and Osteoarthritis Outcome Score for Joint Replacement (KOOS JR) and EuroQol 5-dimension 5-level (EQ-5D-5L), postoperative opioid use to manage knee pain at one- and three-months after operation, and knee-related adverse events. Knee-related adverse events were categorized as deep infection, stiffness, pain, revision, wound complications (including bleeding, delayed healing, hematoma, superficial infection, dehiscence, and drainage), and other knee-related adverse events.

**Fig. 1:**
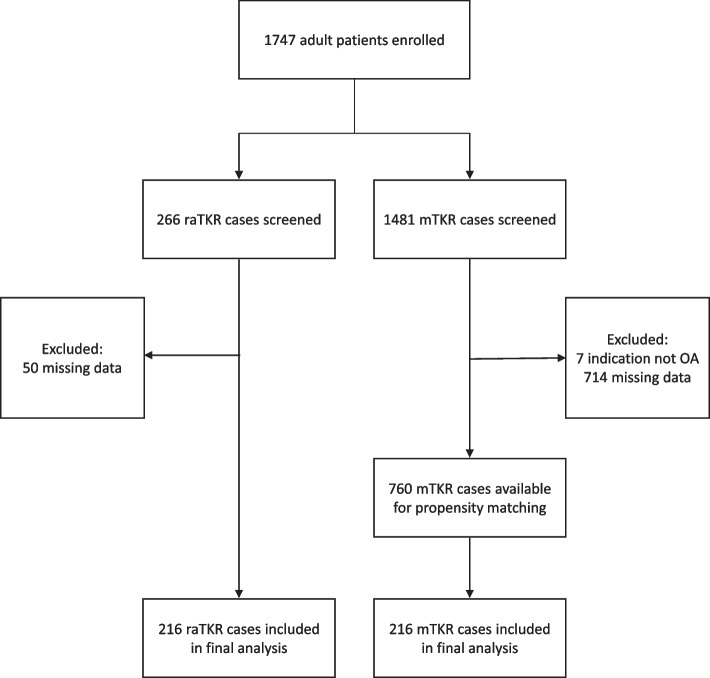
Patient attrition flow chart

To be eligible for inclusion in the study, all patients had to be at least 18 years of age, were scheduled for a unilateral primary TKR indicated due to osteoarthritis, and capable of walking with minimal assistance (a single walking stick or single crutch) preoperatively. Exclusion criteria included (1) substance abuse as determined by the surgeon, (2) inflammatory arthropathies, which would interfere with or compromise activity profiles, (3) those currently undergoing other surgical interventions studies, and (4) those requiring simultaneous or staged bilateral knee arthroplasties less than 90 days apart. If the criteria were satisfied, the informed consent was obtained from patients who elected to proceed were included. For this analysis, all participants in the study who were indicated for raTKR, (*n* = 216) performed between August 2019 and April 2022 using the ROSA^®^ Knee System (Zimmer Biomet, Montreal, QC, Canada), were included for review. Procedures were performed by a total of 46 surgeons, with 31 surgeons performing only mTKR (*n* = 146), 4 surgeons performing only raTKR (*n* = 79) and 11 surgeons performing both mTKR (*n* = 70) and raTKR (*n* = 137). It is important to note that all surgeons were unaware of the fact that comparisons would be made between raTKR and mTKR in terms of the data at the time of data collection. Patients who received less than a three-month follow-up were then excluded (Fig. 1). Additionally, 50 patients were excluded due to missing preoperative or data 1-month after operation. Of the 1,481 participants who underwent mTKR, 216 were matched to the raTKR cases in terms of propensity scores to select matched controls of mTKR from the same database at a 1:1 ratio, on the basis of age, sex, BMI, and comorbidity index. The comorbidity index was aggregated to create a continuous variable (comorbidity index), to be included in multivariate models: congestive heart failure; coronary artery or valve disease; diabetes; chronic pulmonary disease including asthma, chronic bronchitis, COPD or emphysema; dementia or Alzheimer’s disease; previous stroke or transient ischemic attack; muscular dystrophy; previous cervical spinal surgery; previous lumbar spinal surgery; history of cancer; chronic kidney disease; liver disease; rheumatoid arthritis; or paralysis. The Strobe guidelines for reporting of observational studies were followed.

The data were collected on pre- and post-operative case report forms and subsequently monitored for accuracy using random samples both on-site and remotely. Both flexion and extension active and passive ROM values, collected preoperatively through 90-day postoperatively, were reviewed. Flexion values were obtained as the maximum active and passive flexion in degrees. Extension values were recorded in degrees and considered positive for flexion contractures or extension lag (i.e., five degrees of extension is equal to a five-degree flexion contracture or extension lag) and negative for hyperextension. All patients underwent self-directed rehabilitation through the mymobility smartphone-based care management platform (Zimmer Biomet, Warsaw, IN, USA), which provided patients with an at-home based therapy program standard to the surgical institution’s standard of care through the app beginning at discharge through 90-days post-operative. Patients were also prescribed in-patient postoperative physical therapy rehabilitation at their surgeon’s discretion.

Intraoperatively, patients in the raTKR group received either the Persona^®^ Knee System (Zimmer Biomet, Warsaw, IN, USA), Vanguard^®^ Knee System (Zimmer Biomet, Warsaw, IN, USA) or the NexGen^®^ Knee System (Zimmer Biomet, Warsaw, IN, USA). Similarly, patients in the mTKR group received either patients in the raTKR group received either the Persona^®^ Knee System (Zimmer Biomet, Warsaw, IN, USA), Vanguard^®^ Knee System (Zimmer Biomet, Warsaw, IN, USA) NexGen^®^ Knee System (Zimmer Biomet, Warsaw, IN, USA), or the Natural-Knee^®^ System (Zimmer Biomet, Warsaw, IN, USA). The selection of components was left to the surgeons’ discretion. Tibial insert information can be found in Table 1. The patella was resurfaced similarly in the two groups (raTKR 18.1%, mTKR 19.4%, *P* = 0.8054).

**Table 1:** Age, BMI, comorbidity index summarized, and tibial articulating surface by mean ± std (*n*, median, min–max)

Variable	raTKR	mTKR	*t*-Test *P-*Value
Age (years)	62.6 ± 8.12 (216, 63, 43–83)	62.6 ± 8.82 (216, 64, 30–86)	0.9457
BMI^a^ (kg/m^2^)	31.9 ± 6.12 (216, 29.8, 15.6–52.7)	31.7 ± 6.39 (216, 30.9, 18.6–51.7)	0.4022
Derived Comorbidity Index	0.83 ± 1.12 (216, 0.5, 0–6)	0.85 ± 1.12 (216, 0.5, 0–5)	0.6621
Sex *n* = Female (%)	131 (60.6%)	131 (60.6%)	1.0000
ASA Class			0.6218^#^
I	11 (5.2%)	15 (6.9%)	
II	125 (59.0%)	137 (63.4%)	
III	71 (33.5%)	58 (26.9%)	
IV	1 (0.5%)	1 (0.5%)	
Not Reported	4 (1.9%)	5 (2.3%)	
Tibial Articulating Surface			
Cruciate Retaining	11	37	<0.0001^#^
Posterior Stabilized	49	55	
Ultra-Congruent	2	47	
Medial Congruent	120	52	
Constrained Posterior Stabilized	33	17	

^a ^Body Mass Index;* #*: Fisher’s Exact Test

Multivariable longitudinal regression was used to evaluate the difference in active ROM over time, values were reported as least squares mean (95% confidence interval). The longitudinal model tested the treatment effect (raTKR vs. mTKR), time effect, and their interaction with control on the covariance of age, sex, BMI, comorbidities, tibial articulating surface, and preoperative flexion. Sub-group analysis for flexion aROM was performed for patients who received a medial congruent (MC) or posterior-stabilized (PS) tibial component. Logistic regression was employed to analyze the active flexion level at one month (cut by 90°) and three months (cut by 110°) after operation. Statistical analysis was performed using SAS v9.4 (2013, SAS Institute, Inc. Cary, NC, USA) and significance was assessed at *P* < 0.05. Descriptive statistics were used to demonstrate medical events and surgical data, and Fisher’s exact test was utilized to make comparison between groups.

## Results

There was no difference in patient demographics or comorbidity index status at baseline (Table 1).

**Fig. 2:**
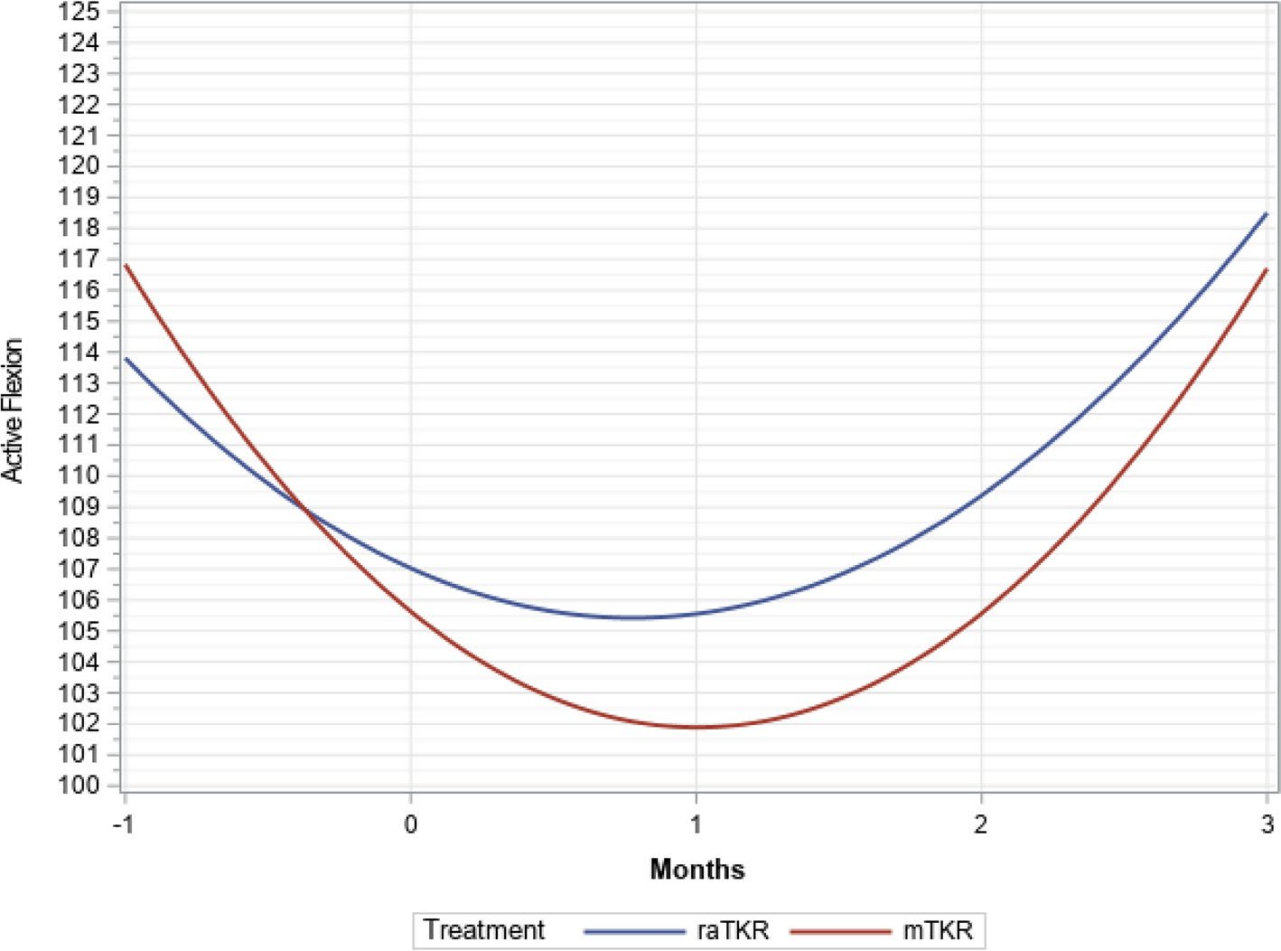
Flexion trend over time (least square means)

**Fig. 3:**
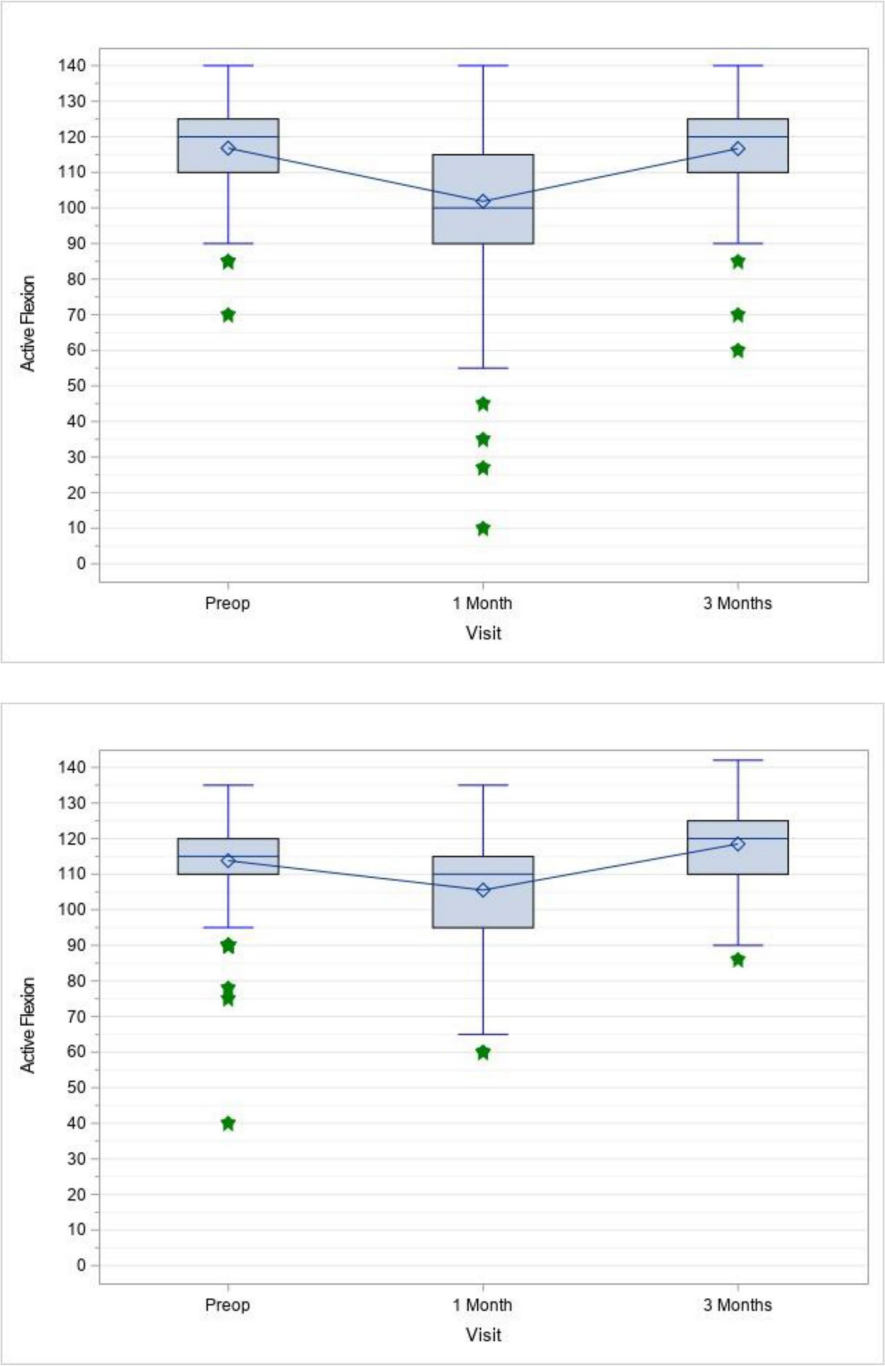
Active flexion box and whiskers plot for manual (Upper) and robotic (Lower) TKR cases

**Fig. 4:**
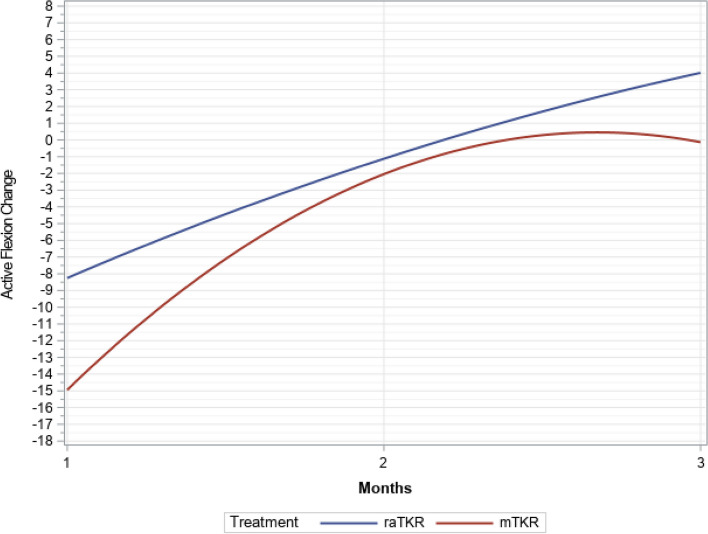
Average active flexion improvement over time (least square means)

There were significant time (*P* < 0.0001), group (*P* < 0.0001), and time by group interactions (*P* < 0.0001), but not group by tibial articulating surface interactions (*P* = 0.6797) found in the multivariate longitudinal model. At one-month and three-months post operation, the raTKR cases had more active ROM for flexion (Figs. 2 and 3) by an average of 5.1° (*P* < 0.001) and 2.9° (*P* = 0.021) (Tables 2 and 3). The raTKR group had a greater improvement (Fig. 4) from preoperative values at both one-month, with an average 6.9° (3.5°, 10.4°, *P* < 0.001) more improvement, and at three-months, with an average improvement of 4.9° more (2.1°, 7.7°, *P* = 0.004). Similar differences were found in passive flexion ROM (Tables 4 and 5). Additionally, the raTKR cohort demonstrated not only a return to preoperative active ROM, but surpassed it at 3 months postoperatively, different from the mTKR cohort (Tables 2 and 3).

**Table 2:** Least squares means difference and comparison for treatment by time interval for active flexion

**Time Interval**		**Estimate** **(Standard Error)**	**95% CI**^a^	**Adjusted** ***P-*Value**
Preoperative	mTKR vs. raTKR	1.58° (1.15°)	-0.68°, 3.85°	0.743
One Month Post Operation	mTKR vs. raTKR	-5.11° (1.15°)	-7.37°, -2.85°	<0.001*
Three Months Post Operation	mTKR vs. raTKR	-2.89° (1.25°)	-5.35°, -0.44°	0.021*

^a ^Confidence interval; *: Statistically Significant

**Table 3:** Least squares means for treatment by time interval for active flexion

**Time Interval**	**Treatment**	**Estimate (Standard Error)**	**95% CI**^a^
Preoperative	raTKR	114.6 (0.82)	113.0, 116.2
	mTKR	116.1 (0.82)	114.6, 117.8
One Month Post Operation	raTKR	106.3 (0.82)	104.7, 107.9
	mTKR	101.2 (0.82)	99.6, 102.8
Three Months Post Operation	raTKR	118.9 (0.95)	117.1, 120.8
	mTKR	116.0 (0.82)	114.4, 117.6

^a^ Confidence interval

**Table 4:** Least squares means difference and comparison for treatment by time interval for passive flexion

**Time Interval**		**Estimate** **(Standard Error)**	**95% CI**^a^	**Adjusted** ***P*-Value**
Preoperative	mTKR vs. raTKR	0.94° (1.13°)	-1.29°, 3.16°	0.408
One Month Post Operation	mTKR vs. raTKR	-5.12° (1.13°)	-7.34°, -2.89°	< 0.001*
Three Months Post Operation	mTKR vs. raTKR	-2.50° (1.23)	-4.91°, -0.10°	0.321

^a^ Confidence interval; *: Statistically Significant

**Table 5:** Least squares means for treatment by time interval for passive flexion

**Time Interval**	**Treatment**	**Estimate (Standard Error)**	**95% CI**^a^
Preoperative	raTKR	116.9 (0.81)	115.3, 118.5
	mTKR	117.8 (0.80)	116.3, 119.4
One Month Post Operation	raTKR	109.0 (0.81)	107.4, 110.6
	mTKR	103.9 (0.80)	102.3, 105.5
Three Months Post Operation	raTKR	121.0 (0.93)	119.7, 122.8
	mTKR	118.5 (0.80)	120.1, 122.8

^a^ Confidence interval

When comparing only cases with an MC bearing and the Persona Knee implant, there were no significant (*P* = 0.310) differences in ROM between subgroups before operation. However, the robotic group showed improved ROM by 5.51° at one-month compared to the manual group (*P* = 0.006). When comparing only cases with a PS bearing, there were no significant differences between sub-groups preoperatively in flexion (*P* = 0.915) or extension (*P* = 0.449). Differences were found between groups at 3 months after operation that significantly favored the raTKR group for active flexion (122.6° ± 8.5° vs. 118.2° ± 10.8°, *P* = 0.043) and active extension (0.2° ± 3.3° vs. 1.6° ± 2.9°, *P* = 0.037). We also found a non-significant (*P* = 0.071) trend for less loss of active flexion between raTKR (-3.6°, 95% CI: -14.4°, 7.2°) and mTKR (-14.3°, 95% CI: -18.9°, -9.81°) in the cruciate retaining sub-group, albeit the sample size was limited.

**Fig. 5:**
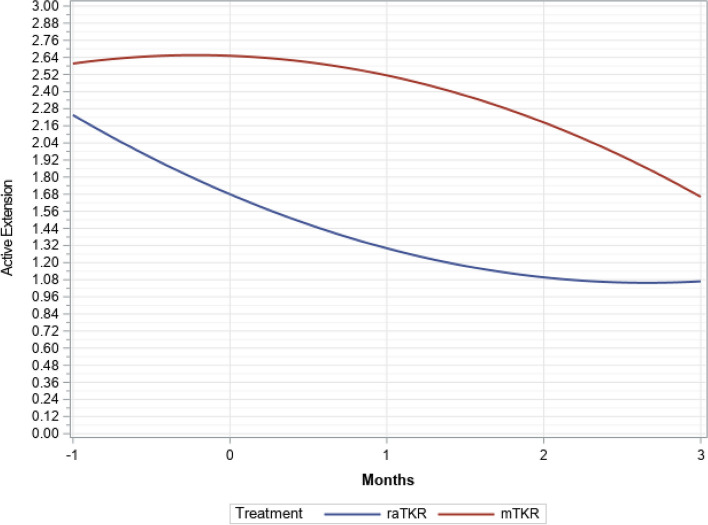
Extension trend over time (least square means)

**Fig. 6:**
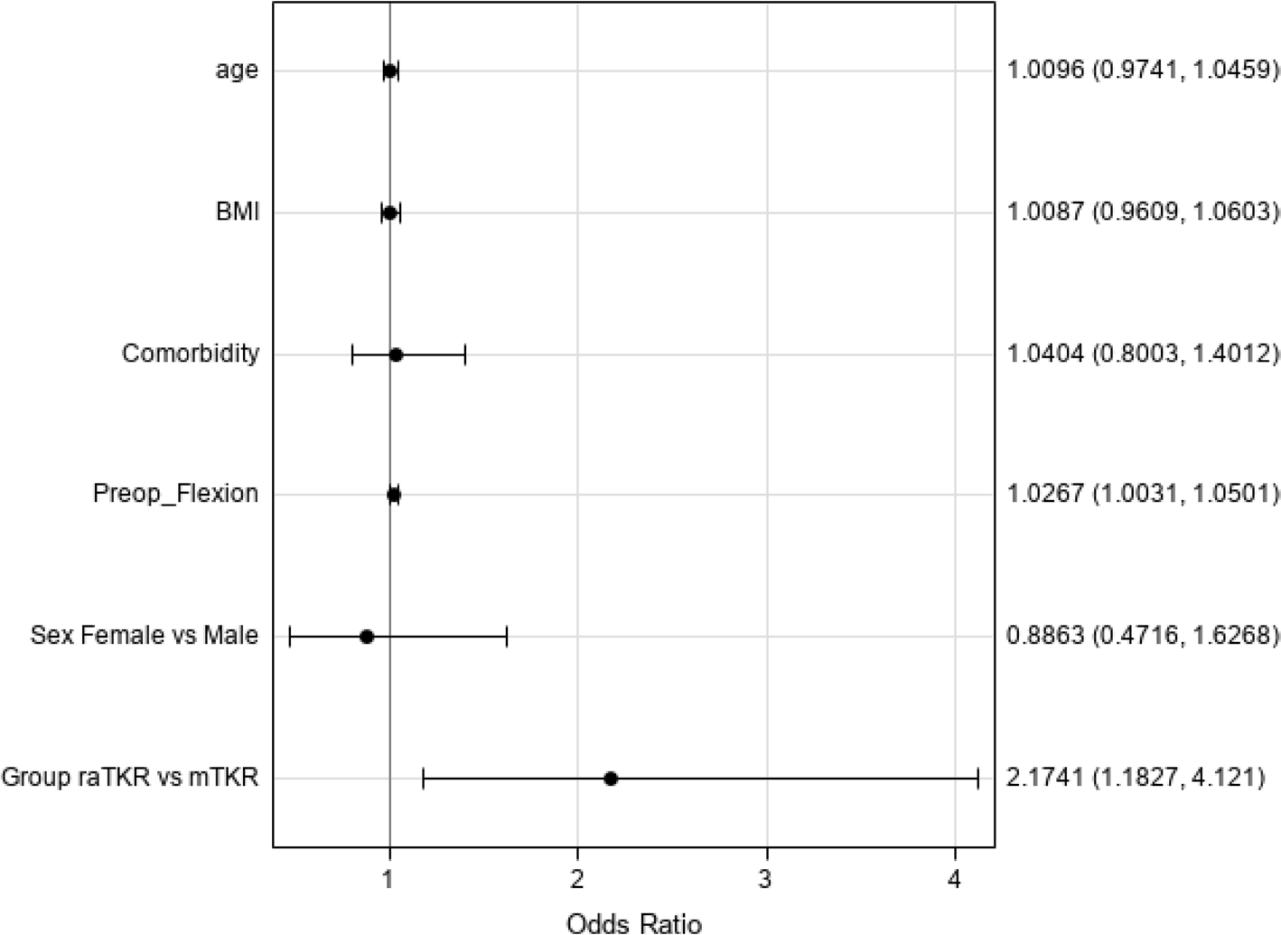
Odds ratio with 95% Wald confidence intervals for achieving ≥ 90° of active flexion at one-month

Active ROM for extension (Fig. 5) was lower overall in the raTKR group by an average of 0.44° (*P* = 0.029). There were no significant (*P* = 0.069) differences in passive extension between groups. The raTKR patients had higher odds of achieving ≥ 90° of flexion at one month (OR 2.15, 95% CI 1.16, 3.99, Fig. 6).

There were no significant (*P* > 0.05) differences between groups at any time point in terms of KOOS JR or EQ-5D-5L (Table 6). There were significant differences between groups for opioid use at one month (mTKR: *n* = 92 (42.6%) vs. raTKR: *n* = 67 (31.2%), *P* = 0.017), but not at three months postoperatively (*P* = 0.703).

**Table 6:** Patient reported outcome measures through one-year follow-up

PROM	raTKR	mTKR	*t*-Test *P*-Value
KOOS JR			
Preoperative	52.18 ± 11.96	51.58 ± 14.11	0.6383
One Month	63.15 ± 10.33	62.94 ± 9.97	0.8326
Three Months	68.9 ± 12.58	70.49 ± 13.18	0.2292
Six Months	73.98 ± 14.12	74.63 ± 13.49	0.6733
One Year	78.61 ± 13.64	79.49 ± 15.7	0.6576
EQ-5D-5L			
Preoperative	0.61 ± 0.23	0.57 ± 0.27	0.0728
One-Month	0.7 ± 0.19	0.67 ± 0.19	0.0872
Three-Months	0.8 ± 0.17	0.8 ± 0.2	0.7703
Six-Months	0.83 ± 0.17	0.81 ± 0.21	0.5392
One-Year	0.86 ± 0.19	0.85 ± 0.19	0.6210

There was no significant (*P* > 0.999) difference in intraoperative complications with (raTKR having 2 cases (0.93%) and mTKR 2 cases (0.93%). There was a total of 69 adverse events in mTKR group and 42 adverse events in raTKR group. There were significantly (*P* = 0.0234) fewer wound complications in the raTKR group, however, no other significant (*P* > 0.05) differences were found in the rates of specific adverse events between groups (Table 7). There were 4 cases of revision in mTKR group and 1 case of revision in raTKR group, and 10 cases of manipulation under anesthesia in mTKR group and 5 cases in raTKR group (Table 8). There was one case of pin site infection in the raTKR group. Other knee-related adverse events included swelling (raTKR: *n* = 9, mTKR: *n* = 5), calf tenderness leading to a negative screening for DVT (raTKR: *n* = 3, mTKR: *n* = 4), iliotibial band weakness or tendonitis (raTKR: *n* = 1, mTKR: *n* = 1), injury during physical activities (raTKR: *n* = 1, mTKR: *n* = 2), and muscle weakness (mTKR: *n* = 1). General anesthesia was used in 136 cases (63%) of raTKR and 114 cases (52.8%) of mTKR (*P* = 0.0406). The length of stay was longer (*P* < 0.0001) in raTKR group (2.7 ± 3.5 days) compared to mTKR group (0.7 ± 1.0 days) and the raTKR group completed more physical therapy visits while in hospital (2.5 ± 2.3 vs. 1.6 ± 1.4, *P* < 0.0001). There was no significant (*P* = 0.6998) difference in the number of patients prescribed physical therapy at discharge (raTKR: 99; mTKR: 104), and the number of patients discharged to skilled nursing facilities (raTKR: 7; mTKR: 6).

**Table 7:** Postoperative adverse events

Adverse Event	raTKR	mTKR	*P*-Value*
Deep Knee Infection	2 (0.9%)	2 (0.9%)	NA
Stiffness	13 (6.0%)	23 (10.6%)	0.0817
Pain	6 (2.8%)	13 (6.0%)	0.1005
Wound Complications	6 (2.8%)	18 (8.3%)	0.0234
Other Knee Related Adverse Events	15 (6.9%)	13 (6.0%)	0.6960

^*^ Chi Square analysis

**Table 8:** Revisions and reoperations

Adverse Events	raTKR	mTKR	*P* value*
Revisions			
Septic	1 (0.5%)	2 (0.9%)	0.5623
Aseptic	0 (0.0%)	2 (0.9%)	NA
Reoperations			
Manipulations under anesthesia	5 (2.3%)	10 (4.6%)	0.1889

^*^ Chi Square analysis

## Discussion

The most important finding of this study was that raTKR attained greater active flexion ROM gains that exceeded preoperative values in the early postoperative period compared to mTKR. The clinical significance of this is limited since the gains was modest. Our results are consistent with two other recent studies that employed the same semi-autonomous robotic system and reported greater passive ROM at one-month [7], 3 months and one year [11] after operation when compared to mTKR and computer-navigated TKR, respectively.

Numerous studies reported an association between 12–24 months postoperative ROM and PROMs [23, 24, 33]. The several studies suggested that patient-reported satisfaction, quality of life, and functional outcomes are most strongly associated with the change in ROM, rather than the absolute ROM achieved [19, 22, 26]. Additionally, patients with a higher degree of preoperative active ROM (>95°) tend to lose active ROM postoperatively [22, 34]. Given that one- to three-month postoperative ROM is predictive of ROM at 12 months [19, 25], our findings of greater changes in active ROM with raTKR at one- and three-months post operation suggest a faster return to activity following primary TKR using robotic assistance. This faster recovery of active ROM may also have economic implications related to reduced knee stiffness. For example, Olsen et al. [35] recently reported stiffness resulted in up to a 7.5-fold greater financial impact, as patients with stiffness required more physical therapy and clinic visits at triple the cost, had a higher revision rate (7.6% vs. 2.8%), and incurred a greater cost per patient both without ($9,401 vs. $5,259) and with ($65,771 vs. $48,287) revision surgery.

In contrast to previous studies that reported greater ROM and KOOS pain and function scores with raTKR [11], this study showed that KOOS-JR scores were not different between groups despite earlier active ROM recovery with raTKR. These findings during the early preoperative period are not unprecedented, as a recent study found no significant correlations between functional, active, or passive ROM with KOOS JR [36]. These findings are neither unexpected, as the KOOR JR questionnaire contains 4 out of 7 questions that directly query pain, 2 questions that indirectly ask about pain, and only one question is related to ROM. Several prior reports demonstrated that the nature of questions on PROMs leads patients to incorrectly conflate reductions in pain and improvements in function [37–41]. However, since the gains in active ROM were clinically modest in the present study, it is also possible they may not have been perceived by patients in the raTKR group during their daily activities.

Two potential mechanisms may explain the faster recovery of active ROM with raTKR than with mTKR. First, excessive tibial slope affects femoral rollback on the tibia, leads to flexion instability, negatively affects ROM and is often under-diagnosed [42, 43]. While we did not assess this, several studies have reported more accurate sagittal tibial resection angles and fewer sagittal outliers with raTKR [16, 17, 44], which may explain the greater active ROM achieved with raTKR. Second, the greater precision of raTKR also leads to less peri-articular soft tissue disruption, inflammatory cytokines and neutrophil infiltration, bone trauma and macroscopic soft tissue injuries, and greater preservation of the peri-articular soft tissue envelope compared to mTKR [17, 45–48]. Unfortunately, this has not yet been studied with the system referenced in this study, and further research is needed to demonstrate this finding. Several studies also reported less early- and mid-term postoperative pain with raTKR as compared to mTKR [10, 11, 27, 48–50]. The severity of one- and five-day postoperative pain was found to be negatively associated with three-month functional outcomes, such as ROM, gait speed, and KOOS scores [51]. In the present study, we found significantly less patients required opioids to treat postoperative pain at one month after operation, and there was a non-significant trend for lower frequencies of pain reported as adverse events with raTKR. Thus, it is plausible that more precise resection, less tissue damage, inflammation, and postoperative pain may explain the faster recovery of active ROM with raTKR observed in this study. Further study is required to assess whether these modest gains improve patient satisfaction, pain, and function over time.

## Limitations

Though the inherent risks of a secondary data analysis include of limited data availability, this prospective study was able to collect sufficient data for this propensity-matched analysis. The primary study data were collected for the purpose of evaluating a smartphone-based care management platform and not specifically to compare robotic vs. manual TKR outcomes. The cohorts were selected from a global multicenter clinical study and may not be homogenous groups. However, these cohorts were selected based on the I/E criteria, and the manual group was propensity-matched to the robotics group to minimize these limitations. Additionally, we were limited to evaluating ROM at 30 and 90 postoperative days. However, since several studies pointed toward faster recovery following raTKR [10, 11, 27, 46], our primary purpose was to confirm these findings with objective results as opposed to PROMs.

The inability to standardize the alignment technique was another limitation of this study. We were also unable to standardize the components implanted between groups, which resulted in differences in the knee prosthesis and tibial polyethylene articulating surfaces between groups. This could be accounted for in those with posterior stabilized (PS) components, but not with medial congruent (MC) components which were significantly more in the raTKR group. Conversely, more mTKR patients received either cruciate retaining (CR) (17.8% vs. 5.1%) or ultra-congruent (UC) (22.6% vs. 1.0%) components compared to raTKR patients. A recent meta-analysis reported a small, but statistically significant, greater postoperative flexion ROM in PS TKR compared to CR TKR [52], and a recent clinical study reported greater postoperative flexion ROM with MC compared to UC [53]. However, subgroup analysis of patients who received an MC component revealed greater aROM flexion in the raTKR group at one month, with differences in ROM similar to the primary analysis (5.51° vs. 5.54°). Subgroup analysis of the PS component revealed similar results, with significantly greater aROM flexion at three months in raTKR group and a similar trend favoring raTKR was found for CR components.

Lastly, there were also differences between groups in anesthesia and length of stay. Significantly more patients in the raTKR group received general anesthesia, which varies between the countries but was associated with greater length of stay [54] and risk of complications [55, 56] following TKR. There were no differences in complications between groups, but the length of stay and the number of in-hospital physical therapy sessions were greater in patients receiving raTKR. This difference is most likely attributable to regional differences in standard of care. A majority of the raTKR operations were performed in Australia, where the standard of care provides for longer postoperative hospital stays (4–5 days on average) and more physical therapy sessions following TKR [57]. However, the difference of one physical therapy sessions between groups, although statistically significant, was likely not clinically meaningful [58–60].

## Conclusion

Robotic-assisted TKR was associated with a lower loss of aROM than mTKR in the immediate postoperative period and significantly higher odds of achieving 90° of flexion within one month postoperatively. Further, on average, raTKR patients exceeded preoperative active ROM within three months after operation, unlike mTKR patients. However, these gains are clinically modest and additional research is necessary to determine if they are associated with component position or less soft-tissue disruption and ultimately improve patient outcomes.

## Data Availability

The data sets generated and/or analyzed during the current study are not publicly available due to proprietary information.
